# Insect Bite Hypersensitivity in Horses: Causes, Diagnosis, Scoring and New Therapies

**DOI:** 10.3390/ani13152514

**Published:** 2023-08-04

**Authors:** Abbey Cox, Allison J. Stewart

**Affiliations:** School of Veterinary Science, University of Queensland Gatton, 5391 Warrego Highway, Gatton, QLD 4343, Australia; a.cox@uq.edu.au

**Keywords:** dermatology, allergy, IBH, Queensland itch, sweet itch, equine summer eczema

## Abstract

**Simple Summary:**

Insect Bite Hypersensitivity (IBH) is the most common cause of itchiness in horses and occurs when individual horses are allergic to the bites of midges or other insects. The itchiness leads to rubbing and often traumatic injury to the skin. There is currently no cure for this disease and management is life-long and costly. This review describes the causes, immune effects, diagnosis, and severity grading as well as traditional treatment and management strategies. Newer treatments for this disease, including topical treatments and vaccination, are also presented.

**Abstract:**

Insect Bite Hypersensitivity (IBH, Queensland itch, sweet itch, equine summer eczema) is the most common pruritic disease of horses. It is most often caused by sensitivity to the saliva of *Culicoides* spp. of biting midges; however, it can also be caused by hypersensitivity to other insect species. The prevalence of IBH in horses is reported to be as high as 60% in some parts of the world. Due to the severe pruritus and effects of secondary self-trauma, IBH has animal welfare concerns, and there is currently no cure. Management of this condition is life-long, time consuming and costly. New grading systems to document disease severity are being validated, which will allow the comparison of clinical trial results of new and existing therapies. Management involves the minimisation of insect bites by use of stabling, fans, rugs and repellents. Symptomatic therapy involves the administration of systemic or topical corticosteroids, systemic antihistamines, and creams and sprays to promote skin healing and decrease inflammation. New immune-mediated therapeutics including vaccines, in addition to desensitisation procedures, show promise at controlling hypersensitivity reactions. This article will review aetiologic agents, pathophysiology, scoring systems and current and new therapies.

## 1. Implicated Insects

IBH is most often caused by hypersensitivity to the salivary proteins of *Culicoides* midges; however, horses can also show hypersensitivity reactions to other insects, including stable flies, mosquitos, black flies, horn flies and tabanids [1]. *Culicoides* midges are more active in warmer temperatures [2,3] and moisture and habitat components determine the presence and activity of the midges [3]. Practically, this means that clinical disease is seen most often in the warmer summer months; however, in severely affected horses or certain geographical locations disease may be seen all year round.

## 2. Distribution and Type of Lesions

**Figure 1:**
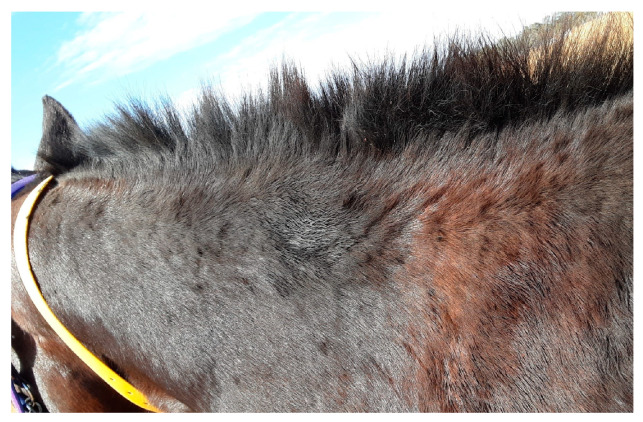
Broken hairs from rubbing secondary to pruritus.

**Figure 2:**
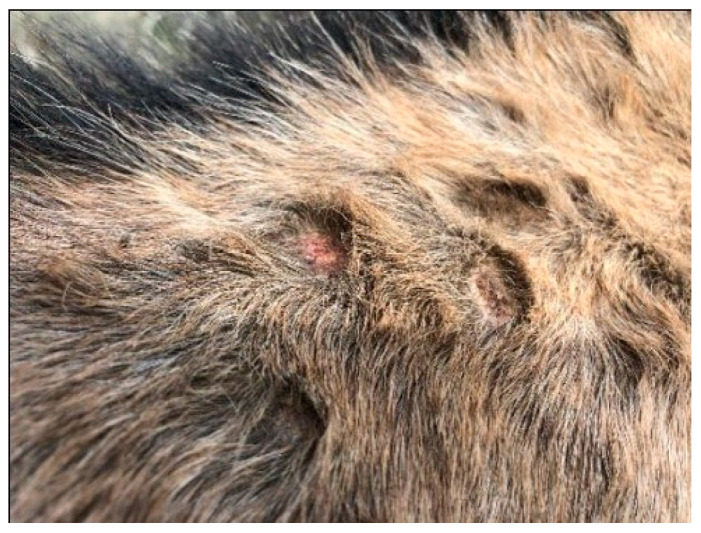
Broken hairs, crusting, alopecia and ulceration from severe pruritus.

**Figure 3:**
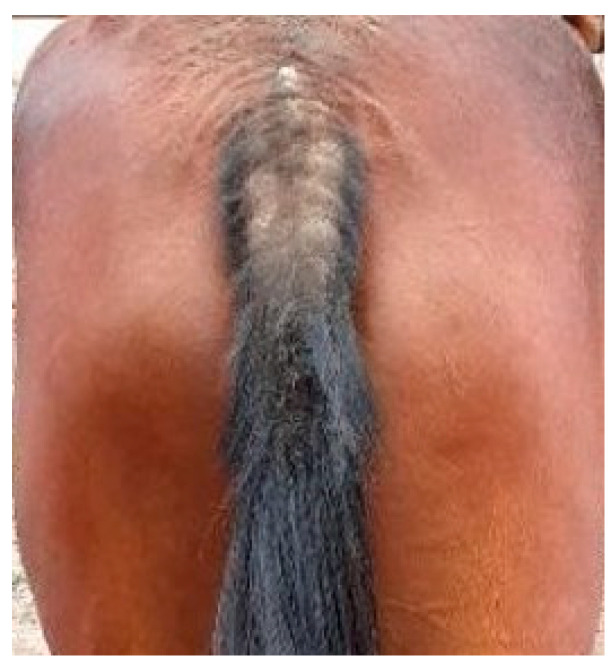
Lichenification and alopecia of the tail.

**Figure 4:**
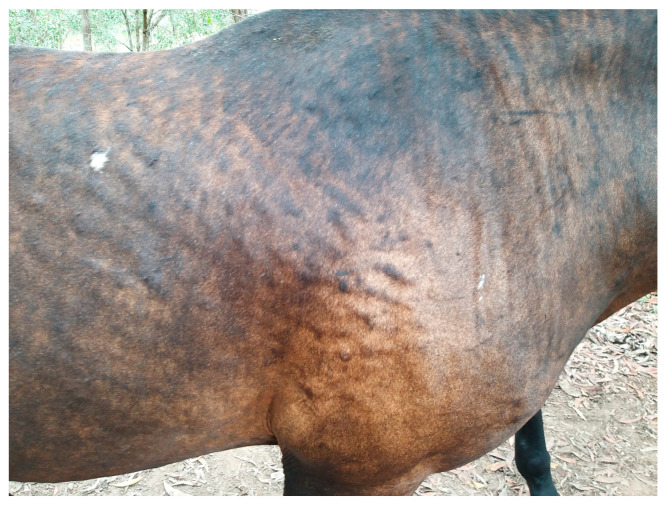
Urticaria.

Lesions caused by IBH are well recognised by horse owners and veterinary practitioners [4]. Bites from the offending insects cause a hypersensitivity reaction which presents clinically as severe pruritus. Most of the lesions characteristic of this disease develop from the self-trauma (scratching and rubbing) inflicted in response to this pruritus [1]. These lesions include alopecia, crusting, ulcers, lichenification and pigmentary alterations [1,5] (Figure 1, Figure 2 and Figure 3). Lesions can be severe with oozing, bleeding and purulence from secondary bacterial and yeast infections. Secondary infections in addition to a more pervious skin barrier may also contribute to the continued pruritus [1]. Urticaria may also be present [5] (Figure 4). In contrast to the characteristic lesions that occur secondary to the intense pruritus, urticaria is a primary lesion caused by the degranulation of dermal mast and other cells releasing histamine, platelet-activating factors and prostaglandins [6]. Horses can also develop behavioural changes in response to persistent pruritus and lesions [1,5].

The distribution of IBH lesions on affected horses are variable depending on the species of midge involved [1,5,7,8]. Lesions are commonly found on the dorsal part of the body, affecting the mane, withers and tail, as well as the ventral part of the body [9]. It is the authors observation that ventral lesions are comparatively rare in Australia, yet are reported more commonly in other parts of the world. If several species of *Culicoides* are present in a geographical area, horses may show a combined distribution of clinical signs or varying distribution of clinical signs at different times of the year, depending on the seasonality of the offending midges [1,8]. Most horses begin to show signs around 3–4 years of age, with clinical signs worsening with age. There is no breed, colour or sex predisposition [5,8], although there is a recognised familial component in some populations [9].

The *Culicoides* midge is not present in Iceland and, thus, local horses are not affected by IBH due to absence of antigenic challenge [10]. In one study, Icelandic horses exported to *Culicoides*-endemic countries had an average incidence of IBH of 34.5% and an incidence as high as 49.5% for those exported more than 2 years prior [2]. The risk of IBH development is also known to increase with age at the time of export [11], likely due to the lack of development of immunologic tolerance with early antigen exposure [12]. Additionally, genetic selection in Europe could potentially exclude horses with clinical signs of IBH from breeding programs. This population of horses presents an ideal subset of the equine population in which to study pathophysiology and potential treatments for the disease due to their high genetic predisposition [10,11,13,14,15,16,17].

## 3. Pathophysiology

Research into the pathophysiology of a number of allergic diseases, including IBH, comes from studies in mice, humans and dogs, in addition to work conducted specifically in horses [1]. The pathogenesis of atopic diseases in all species has been demonstrated to be much more complex than previously thought [1].

IBH is caused by the bite (saliva) of insects, most often from *Culicoides* midges [1,12,15,18]. It causes an IgE-mediated, type I (immediate hypersensitivity/anaphylactic reaction), and then type IV (cell-mediated/delayed) hypersensitivity with accompanying eosinophil infiltration into the skin [1,12,15,18]. Eosinophils are not normally present in healthy dermis and their presence is associated with allergy, asthma and parasitic infection [18,19,20].

The mechanisms of *Culicoides* hypersensitivity in horses are complex, and should not be extrapolated directly from other species. There is a mix of Type I and Type IVb hypersensitivity reactions. Type I hypersensitivity is an immediate reaction involving IgE release, mast cell degranulation and the release of histamine and other inflammatory mediators. However, horses have a poor clinical response to antihistamines [21]. Type IV hypersensitivity involves a cell-mediated reaction that requires previous exposure to antigens. As it is difficult to determine if the observed eosinophilia is a result of late-phase Type I or by Type IVb hypersensitivity, it may be simpler to describe the process in terms of a shift from an early IgE-dominated phase to a later phase dominated by eosinophilia [22]. The proposed immunopathogenesis of *Culicoides* hypersensitivity in horses has been recently reviewed in detail [18]. In summary, after a horse is bitten by an offending midge, the allergenic proteins are transported to the regional lymph node. Naïve T-lymphocytes are activated in an initial sensitisation phase. After each subsequent exposure to specific midge allergens, there is an increase in IgE production and eosinophilia [1]. During the early phase of the allergic response, the immune reaction is skewed towards a Th-2 response with the secretion of IL-4 and IL-13, resulting in a traditional IgE-mediated Type I hypersensitivity reaction with the secretion of *Culicoides*-specific IgE and subsequent mast cell and basophil degranulation with the release of histamine [12,23]. After repeated antibody exposure, there is differentiation of conventional Th-2 cells into pathogenic effector Th-2 cells which secrete IL-5, leading to eosinophilia. Pruritus develops due to interactions between the immune and nervous systems attributed to IL-31. The binding of IL-31 to its receptor on sensory neurons stimulates the nerve leading to the sensation of pruritus [1]. Repeated exposure to midge saliva leads to an amplification of the allergic response locally [1]. Chronic allergic disease trends more towards a Th-1 cytokine response, with TNF-α playing a prominent role [1]. There is a resulting characteristic imbalance between the Th-2 and Th-1 lymphocytes [24].

All horses exposed to the saliva of midges will generate a serum antibody response, regardless of whether they suffer from Insect Bite Hypersensitivity or not [25]. Antibodies to these midges are not found in samples from horse populations residing in *Culicoides*-free environments. Only horses showing clinical signs of IBH have IgE antibodies, whereas IgG antibodies are found in all horses exposed to midges, and not just those who develop the more severe allergic reaction [25].

Horses with IBH have been found to have impairment of the epithelial barrier, which can predispose them to the development of IBH [26,27]. Keratinocytes also contribute to the innate immune response, which may also play a role in the pathogenesis of IBH [27].

Immunotherapies, particularly those targeting interleukins IL-31 as well as IL-4, IL-5, IL-6 and IL-13, present potential immunological targets in the control of this disease.

## 4. Diagnosis

Diagnosis of the disease has traditionally been by seasonal history, clinical signs and response to insect control [1]. Other tests with high sensitivity and specificity for IBH have not yet been developed [18]. Histological examinations of lesions may support the diagnosis but are not pathognomonic for the disease. Intradermal and serum ELISA testing can also be used to select the allergens for immunotherapy, but cannot be used as a diagnostic test of the disease due to its low sensitivity and specificity [18].

### 4.1. Intradermal Skin and Serum Allergy Testing

The identification of allergen-specific IgE against the saliva of the *Culicoides* midge forms the basis of intradermal skin testing and also serum allergy testing [1]. The management of IBH with desensitisation immunotherapy fails in some cases, likely due to different species of *Culicoides* present in different regions [1,8].

Studies conducted in a Dutch population of horses [28,29] have shown some promise in the diagnosis of IBH using ELISA and histamine release testing (HRT), with the latter proving to be a more sensitive test. However, histamine release testing is more complex to perform than ELISA, resulting in a less practical test. There have been differences in the accuracy of ELISA tests depending on whether the species of *Culicoides* used to generate test antigens was similar to those residing in the geographic region in which the ELISA was being used to diagnose IBH.

Recent research conducted on serum allergen testing [30] is showing more promise, both in the diagnosis of IBH and also in the development of patient-specific immunotherapy. By using a combination of 7 allergens (out of 27 tested) researchers were able to diagnose > 90% of IBH-affected horses with a specificity of >95%. There is scope for the further development of serum allergen testing, but due to the variation in the species of midges responsible for IBH in various parts of the world, regionally specific *Culicoides* antigen panels would be required to diagnose IBH and identify species-specific antigens to be incorporated into individually tailored immunotherapy.

### 4.2. Genetic Testing of Horses

Genetics and environment both play a role in the susceptibility to allergic disease such as IBH [1]. The inheritance of genes involved in the innate and acquired immune response as well as the structure and function of the skin likely influence which horses are more predisposed to developing clinical signs of allergic skin diseases [1]. Certain breeds of horses appear to be more at risk of developing IBH signs, including Icelandic, Friesian, Arabian, Quarter horses [3,5,8,31], Welsh ponies [8] and Shetland ponies [3]. In the Netherlands, Friesian mares had a much higher prevalence of IBH than Shetland mares [3]. It appears that certain lineages are more susceptible to the disease, rather than outright breeds, and the prevalence of disease in certain breeds is variable in different parts of the world. Further understanding of the genetic influence of IBH may lead to selective breeding or the instigation of preventative strategies, such as relocation, in addition to treatments based on specific genetic traits [9,32].

### 4.3. Scoring the Severity of IBH

There are a wide variety of different protocols used to assess the presence and distribution of lesions, as well as their severity, when examining horses with IBH. Assessment of the severity of pruritus by horse owners has been performed using a 1–10 grading scale, with 1 being very mild and 10 being extremely severe pruritus [33]. Photographs and charts may be used as a more objective way to document and measure the distribution and severity of lesions. Existing scales extrapolated from other species, for example, the psoriasis area and severity index, have also been used [15].

Scoring systems have been validated for dermatologic conditions in humans [34,35] and dogs [36,37,38] and recently in horses using multiple scorers [13]. More detailed scoring of IBH clinical signs can be achieved through dividing the horse into various body parts (head, neck, tail, etc.) and grading various signs based on their severity. The final total score is then calculated based on multiple scores assigned to various signs and their severity in one or more parts of the body [15]. Geiben proposed the use of a grid score for the objective evaluation of improvement in clinical signs in horses in response to treatment in her dissertation on studies into summer eczema and the testing of an immunomodulatory treatment [39].

Three scoring systems derived from previous work on allergic skin conditions have been evaluated by Miller et al. [13]. Using a single scorer, there were strong correlations (Spearman’s rho calculations) between all three scoring systems over the duration of the trial. There was also substantial agreement (Cohen’s kappa) between the scoring systems at the different assessment time points. Ideally, all future clinical trials should be performed using a single validated scoring system that is easy to use, accurate and shows consistent results between numerous scorers [40].

## 5. Management

The management of IBH is currently an expanding field with recent and ongoing research adding to the literature [23]. There is currently no satisfactory curative treatment for this condition [7,12,18].

### 5.1. Traditional Treatments

Management of IBH involves limiting exposure to the *Culicoides* midges and minimising the pruritus to prevent secondary self-trauma. For horses affected by this disease, the only current method to eliminate signs of the disease is for the horse to reside in a *Culicoides*-free environment.

There are commonly recommended strategies for the management of IBH in horses in environments where midges are present [7]. These include

- Minimising exposure to the *Culicoides* midge—this is considered the most important control measure and includes rugging, stabling at dawn and dusk, and the use of fans. It has been shown that horses affected by IBH show more itching behaviours (when compared with unaffected controls) in the evening when midges are most likely to be feeding [41].
- Use of topical repellents [41,42].
- Reduction in disease severity through the use of corticosteroids. Due to potential complications with the long-term use of corticosteroids in horses, they should only be used as a short-term treatment tool [7,33,43,44] and ideally after testing for insulin dysregulation [45].
  ○ The authors have used the following protocol with success: dexamethasone at 0.04 mg/kg IM initially, then 0.03 mg/kg IM for 3 days, then 0.02 mg/kg IM for 14 days, then 0.02 mg/kg IM every other day as required. If there is no initial reduction in pruritus after the initial 5 days (and no evidence of insulin dysregulation), then higher dosages can be trialled. The authors recommend avoiding dosages greater than 0.1 mg/kg IM for more than 5 days.
  ○ The injectable dexamethasone solution can be given orally with a bioavailability of between 28–66% [46]. An intramuscular dosage of 20 mg for a 500 kg horse would be approximately 42 mg orally, assuming an average bioavailability of 47%.

Antihistamines and desensitisation therapies have been trialled in horses with IBH, as they have shown to be effective in other disease process and other species; however, they have not proven to be particularly effective in horses [21].

Due to the severe nature of the disease and the difficulties that owners have in managing the clinical signs, new treatments are being sought for the treatment and management of IBH.

### 5.2. Whole-Pathogen Immunotherapy

The administration of pathogen extracts for the purposes of generating an immune response is not new technology and has been used for the prevention of dermatological diseases in various species [47]. Whole-pathogen immunotherapy was trialled for the preventative management of IBH in a US horse population in 1990 [48] and in British Colombia in 1996 [49]. The administration of a crude *Culicoides* extract resulted in an improvement in trial horses from British Colombia; however, in the US study, a significant response to treatment was not found.

The efficacy of a whole-body extract of the *Culicoides* midge versus placebo found no difference in response to treatment [47]. Allergen-specific immunotherapy has been more successful when deployed locally, as different species of midges exist and allergens from one species may not induce immunity to regionally diverse species of midges. Further development of this technology is required utilising the local species of midges.

### 5.3. Allergen-Specific Immunotherapy

Several *Culicoides* antigens have been produced as recombinant proteins [30], which may be useful for the development of allergen-specific immunotherapy for IBH. *Culicoides*-recombinant R-allergens have been studied in Icelandic horses [50]. As expected, horses became sensitised to *Culicoides* R-antigens within the first year of injection. There was co-sensitisation to IgE reactivity of multiple Culicoides antigens.

The administration of r-*Culicoides* allergens in adjuvant has also been investigated [51,52]. Intralymphatic administration of the allergen caused a high IgG antibody level and immune response in the test population [51]. Vaccination by intralymphatic versus intramuscular injection has also been investigated using this allergen [52]. The test horse population was vaccinated three times (weeks 0, 4 and 8) either by intralymphatic or intramuscular injection of a purified allergen. No difference in immune response between the methods was detected; therefore, intramuscular administration was recommended as a simpler administration method for future work on this technology.

The use of systemically and even orally administered transgenic barley expressing a *Culicoides* antigen induced a specific IgG antibody response in horses not previously exposed to *Culicoides*. This IgG response is thought to partially prevent IgE binding to the allergen, thus decreasing the acute allergic response [14,53]. As these studies involved small numbers of horses, further research is required in larger numbers of IBH-affected horses to test the effectiveness of these antigens in the potential control of IBH.

These sensitising allergens may be useful for preventative allergen immunotherapy in the future.

### 5.4. Cytokine Vaccination

Desensitising vaccines using whole-pathogen immunotherapy for IBH have been available for some time; however, their effectiveness has previously been poor [7]. Knowledge of the pathophysiology of the disease has significantly increased over the past few years, leading to new vaccination strategies. Vaccination still represents the most promising tool currently being explored for the treatment of this disease. Due to the sheer size of the horse, not all vaccination technologies are economically viable. There are several recent publications evaluating the efficacy of vaccinations against IBH [15,16,17,54]. The vaccinations were developed using genetically sequenced equine interleukin proteins coupled to a virus-like particle derived from a cucumber mosaic virus containing tetanus toxoid universal T-cell epitope [15,16,17,54].

#### 5.4.1. Virus-like Particle (VLP)-Based Therapeutic Vaccines Targeting IL-5

Interleukin-5 is the cytokine responsible for the maturation of eosinophils in the bone marrow and eosinophil release into the circulatory system in response to allogenic antigens. An innovative approach utilising a virus-like particle-based therapeutic vaccination against IL-5 was developed to induce auto-antibodies to decrease eosinophil production and dermal migration, thus minimising tissue inflammation and damage [15]. In a double-blinded, placebo-controlled randomised study, horses were administered the vaccination (or placebo) monthly for 3 months and then again 2 months later. Clinical improvement, antibody titres, blood eosinophil counts and parasite load through faecal floats were measured for the duration of the study. A 50% or greater reduction in clinical signs was observed in 47% of vaccinated horses and only 13% of unvaccinated horses (*p* < 0.05). A 75% reduction in clinical signs was observed in 21% of vaccinated horses and none of the unvaccinated group (*p* < 0.05). An increase in antibody titres against IL-5 were also detected in 17/19 vaccinated horses [15].

Thirty of these horses were monitored over a longer duration [16]. Antibody levels were further increased with corresponding clinical improvement after the 2nd year of vaccination compared to placebo-treated horses.

Another study also showed decreased basophil counts along with the expected reduction in blood eosinophils [54]. Decreased basophil counts were not identified after the first vaccination year; however, they were evident after the second.

No adverse effects of vaccination were noted in any of the conducted studies [15,16,54]. This was supported by a further study, thus demonstrating that the IL-5 vaccine has been shown to be a safe therapeutic option for the treatment of IBH [55]. Yearly vaccination against IL-5 could be used as a potential tool for longer-term control of the clinical signs associated with IBH.

#### 5.4.2. Virus-like Particle (VLP)-Based Therapeutic Vaccines Targeting IL-31

Vaccination against IL-31 was assessed for its effectiveness in reducing pruritus in horses with IBH [17]. The horses were examined by a blinded assessor over a 2-year period following a protocol similar to Fettelschloss-Gabriel et al. [15]. The vaccinated horses showed clinical improvement over the course of the trial. This study demonstrated the involvement of IL-31 in the pathophysiology of IBH and provided another treatment arm in the management and possible control of IBH.

Currently, the large size and weight of horses precludes the economical use of virus-like particle (VLP)-based therapeutic vaccines targeting IL-5 and 31 in horses [18,56]. The next step now in exploring this technology would involve in vivo studies on affected horses.

### 5.5. Topical Treatments

Almost all owners of horses with skin conditions will try various over-the-counter treatments for the management of clinical signs [33]. There are several substances and topical formulations that are anecdotally reported to reduce pruritus and the severity of skin lesions, both in horses and other species. These substances may have soothing and insect-repellent effects. Topical treatments have been trialled in horses with IBH. A cream containing omega-3 fatty acids, humectants and emollients improved skin lesions of affected horses, but had minimal reduction in the control of the underlying pruritus [57]. A double-blinded, placebo-controlled, randomised cross-over Australian study evaluated the efficacy of a purpose-made herbal spray (Red Healer Equine and Canine Spray, NSW, Australia) containing various natural ingredients including *Cinnamonum camphora* (Camphor), *Cymbopogon citratus* (Lemongrass), *Litsea cubeba* (May Chang), *Mentha piperita* (Peppermint), and *Pogostemon cablin* (Patchouli) [42]. These essential oils are reported to have mast cell stabilisation, antipruritic, anti-inflammatory, analgesic and insect-repellent effects [42,58,59,60,61,62,63,64,65]. This study focused on evaluating changes in clinical signs (rather than the mechanism of action of the product) and an improvement in pruritus and disease severity was reported in 95% of treated horses [42]. A recent study analysed a cattle-derived unsaturated aldehyde as a *Culicoides* repellent; however, its efficacy could not be proven [41].

### 5.6. Other Treatments

Oclacitinib (Apoquel^®^), a Janus Kinase inhibitor, has shown to be effective in managing allergic conditions in other species [66,67]. It affects the transduction of IL-31 leading to a decrease in its pruritic effects [66]. This medication has been reportedly used for the management of IBH and other allergies in horses [5]. As the efficacy and safety of oclacitinib in the horse has not yet been determined, further research should be conducted before its use can be recommended.

## 6. Conclusions

Although management strategies for IBH are well understood, client compliance and success is limited. There are many potential avenues for further research in the management of clinical signs of this condition and a multi-modal response may be beneficial. Further work is also required into the validation of a scoring system that can be used in future clinical work.

## Data Availability

Not applicable.
